# Deciphering the Electronic Transitions of Thiophene‐Based Donor‐Acceptor‐Donor Pentameric Ligands Utilized for Multimodal Fluorescence Microscopy of Protein Aggregates

**DOI:** 10.1002/cphc.202000669

**Published:** 2020-12-23

**Authors:** Camilla Gustafsson, Hamid Shirani, Petter Leira, Dirk R. Rehn, Mathieu Linares, K. Peter R. Nilsson, Patrick Norman, Mikael Lindgren

**Affiliations:** ^1^ Department of Physics-Faculty of Natural Sciences Norwegian University of Science and Technology (NTNU) 7491 Trondheim Norway; ^2^ Department of Theoretical Chemistry and Biology School of Engineering Sciences in Chemistry, Biotechnology and Health KTH Royal Institute of Technology 106 91 Stockholm Sweden; ^3^ Division of Chemistry, Department of Physics Chemistry and Biology Linköping University 581 83 Linköping Sweden; ^4^ Laboratory of Organic Electronics and Scientific Visualization Group ITN and Swedish e-Science Research Center (SeRC) Linköping University 581 83 Linköping Sweden

**Keywords:** amyloid detection, fluorescent biomarkers, luminescent conjugated oligothiophenes, solvent effects, spectroscopy calculations

## Abstract

Anionic pentameric thiophene acetates can be used for fluorescence detection and diagnosis of protein amyloid aggregates. Replacing the central thiophene unit by benzothiadiazole (BTD) or quinoxaline (QX) leads to large emission shifts and basic spectral features have been reported [*Chem. Eur. J*. **2015**, *21*, 15133‐13137]. Here we present new detailed experimental results of solvent effects, time‐resolved fluorescence and examples employing multi‐photon microscopy and lifetime imaging. Quantum chemical response calculations elucidate how the introduction of the BTD/QX groups changes the electronic states and emissions. The dramatic red‐shift follows an increased conjugation and quinoid character of the π‐electrons of the thiophene backbone. An efficient charge transfer in the excited states S_1_ and S_2_ compared to the all‐thiophene analogue makes these more sensitive to the polarity and quenching by the solvent. Taken together, the results guide in the interpretation of images of stained Alzheimer disease brain sections employing advanced fluorescence microscopy and lifetime imaging, and can aid in optimizing future fluorescent ligand development.

## Graphical abstract

**Fluorescent biomarkers**: The introduction of donor‐acceptor‐donor moieties in luminescent conjugated oligothiophenes gives a drastic shift in spectroscopic profile. This opens up for designing novel fluorescent biomarkers that can classify different types of amyloid folds and differentiate different morphological protein topologies.

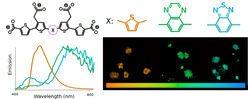

## 1 Introduction

**Scheme 1:**
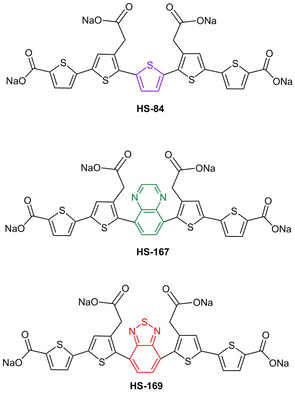
Chemical structure of the thiophene‐based pentameric ligands. All the ligands have a backbone of thiophene rings with different central units. HS‐84 is a pentameric oligothiophene, whereas HS‐167 and HS‐169 have a central QX (green) or BTD (red) moiety, respectively.

Neurodegenerative diseases, like the Alzheimer's disease (AD), are associated with the formation of protein aggregates known as amyloid.^[1]^ The presence and morphology of such protein aggregates can be studied using advanced ultra‐resolution techniques such as AFM, EM and X‐ray diffraction,^[2]^ but also highly sensitive spectroscopic techniques in combination with fluorescence microscopy.^[3]^ Traditionally, the presence of amyloid deposits is readily verified by employing the sensitive fluorescence techniques by staining sections or *in vitro* systems with Congo Red or Thioflavin T (ThT).^[4]^ However, these probes, as well as antibody stains such as 6E10,^[5]^ give little information to identify what type or fold a particular amyloid deposit belongs to. In this context the recently developed luminescent conjugated poly‐ or oligothiophenes (LCP/LCO) have shown promising potential.^[6]^ These can also be investigated with multiphoton excitation spectroscopy.^[7]^ Using double staining it was recently shown that LCOs can distinguish age differences in amyloid plaques in AD mouse models.^[8]^ More recently, thiophene‐based pentameric ligands with donor−acceptor−donor (D−A−D) type electronic structures based on benzothiadiazole (BTD) and quinoxaline (QX) as the central heterocyclic moiety were used to identify a variety of amyloids and carbohydrates. Specifically, the HS‐167 and HS‐169 (Scheme 1) pentameric ligands, where thiophene units act as donors and quinoxaline (QX) or 2,1,3‐benzothiadiazole (BTD) as acceptors, readily identified recombinant Aβ1‐42 fibrils and immunopositive amyloid‐β (Aβ) pathology in human AD brains.^[9]^ HS‐169 has also been utilized for *in vivo* detection of Aβ aggregates in transgenic mice,^[10]^ as well as alpha‐synuclein aggregates isolated from cerebrospinal fluid or brain.^[11]^ Furthermore, HS‐169 was recently employed for spectral assignment of distinct carbohydrates in plant tissue.^[12]^ This assignment could not be afforded by the corresponding pentameric oligothiophene, HS‐84 (Scheme 1), verifying that the D−A−D electronic structure of HS‐169 was essential for distinguishing distinct carbohydrates. Hence, compared to the genuine oligothiophene counterpart, thiophene‐based D−A−D pentameric ligands seems to exhibit additional optical modes for spectral assignment of distinct biomolecular entities. Upon binding to Aβ fibrils there was also a dramatic increase in brightness of the emission bands as compared to the emission in solution. For HS‐167 and HS‐169 the bands were also shifted with approximately 50 nm compared to the full thiophene ligand when binding to the amyloid fibrils.^[9]^

In this report, we examine the detailed photophysical properties of thiophene‐based D−A−D pentameric ligands by using different solvents and both time and spectrally resolved methods, including the characterization of protein aggregates with advanced microscopic modes of detection, such as two‐photon absorption (TPA)^[6b]^ and fluorescence lifetime imaging microscopy (FLIM).^[13]^ Furthermore, the ligands are also studied using quantum mechanical (QM) calculations in order to assess the impact of the molecular geometry on the spectral response.

The most favorable conformation of each ligand was determined and calculations of the fluorescence upon one‐ and two‐photon excitations were performed with the time‐dependent DFT method in order to elucidate which electronic states are involved in the electronic transitions, guiding in the interpretation of experimental spectra. The planarity of the conjugated π‐system in the thiophene rings is associated with a spectroscopic shift in the dominant absorption peak^[14]^ making these ligands suitable for distinguishing different types of disease‐associated protein topologies. Taken together, our multidisciplinary study decipher in detail the photophysical characteristics of D−A−D pentameric oligothiophenes and we foresee that our findings will assist in developing novel ligands that can be utilized for multimodal fluorescent assignment of a variety of disease‐associated protein aggregates.

## 2 Results and Discussion

The preparation of the fluorescent ligands HS‐84, HS‐167 and HS‐169 was previously reported along with their basic excitation and emission properties in PBS as well as mixed with amyloid fibrils.^[9]^ Here the ligands are further characterized using several solvents and employing a time‐resolved mode of detection. The experimental results form the basis for the theoretical investigation to be presented and discussed in more detail in subsections to appear after the presentation of new experimental results.

### 2.1 Absorption and Emission Spectra

**Figure 1:**
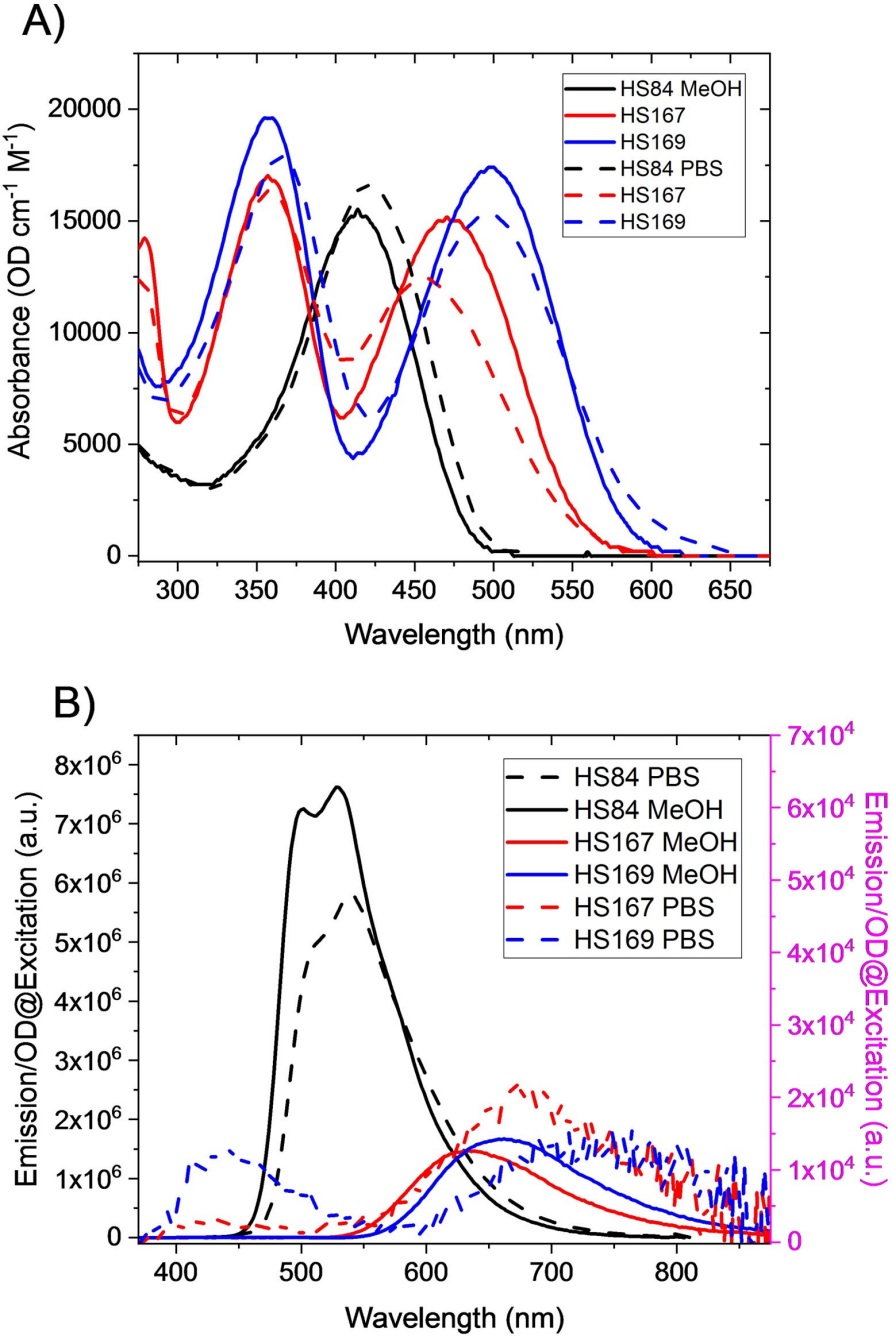
A) Absorbance spectra of HS‐84, HS‐167 and HS‐169 in MeOH (MeOH: solid lines) and PBS (dashed lines). B) Emission spectra of solutes in (A) excited normalized to absorbance (OD) at the excitation wavelength. HS‐167/HS‐169: λ_ex_=350 nm; HS‐84: λ_ex_=400 nm. All samples have a concentration of 5 μM. Note that HS‐167 and HS‐169 in PBS are plotted with the right scale‐bar.

For the sake of elucidating more knowledge of the excited states, we here examine the ligands using several different solvents: methanol (MeOH) and ethanol (EtOH), in addition to PBS (at pH 7.4), the latter usually used in staining sections and studies of *in vitro* amyloid systems. For clarity, the absorption and emission spectra for the three ligands in only PBS and MeOH, are shown in Figure 1. EtOH gave similar results as MeOH, and the photophysical parameters for all three solvents are summarized in Table 1.

**Table 1:** Photophysical parameters for the three ligands in PBS, methanol (MeOH) and ethanol (EtOH).

Sample	λ_abs_ (nm)	λ_em_ (nm)	QE (%)	τ (ns)
HS‐84: PBS	422	538	26±2^[c]^	0.725±0.007^[e]^
MeOH	414	528	30±2^[c]^	0.708±0.007^[e]^
EtOH	414	530	34±2^[f]^	n. d.
				
HS‐167: PBS	359 456	425 675	0.15±0.01^[d]^ –	– 0.033±0.011^[a]^
MeOH	356 470	– 635	5.5±0.5^[d]^ 13±2^[f]^	1.04±0.013^[b]^ 1.19±0.014^[a]^
EtOH	358 477	– 630	8.3±0.6^[d]^ –	1.23±0.014^[b]^ 1.76±0.024^[a]^
				
HS‐169: PBS	365 497	450 725	0.16±0.01^[d]^ –	– 0.044±0.004^[a]^
MeOH	358 498	– 662	10±0.8^[d]^ 12±2^[f]^	1.97±0.013^[b]^ 1.69±0.019^[a]^
EtOH	358 504	– 660	15±0.8^[d]^ –	– 2.44±0.022^[a]^

[a] λ_ex_ : λ_em_=469 : 650 nm. [b] λ_ex_ : λ_em_=337 : 650 nm. [c] λ_ex_=470 nm vs. Coumarin 153 (EtOH): QE=0.544±0.029. [d] λ_ex_=350 nm vs. Coumarin 102 (EtOH): QE=0.764±0.041. [e] λ_ex_ : λ_em_=443 : 530 nm. [f] λ_ex_=450 nm vs. Coumarin 153 (MeOH): QE=0.39±0.03. A GG055 LP (390 nm) filter was used when appropriate to block scattered excitation light.

Using more nonpolar aprotic solvents such as tetrahydrofuran or acetonitrile, there was a substantial broadening of both the absorption and emission bands for all solutes and the emissions of particularly HS‐84 was extremely weakened, along with a red‐shift of approximately 100 nm (data not shown). This might indicate aggregation and poorer solubility, so further experiments with more nonpolar aprotic solvents were not carried out.

The absorption spectra are similar in strength with maximum extinction coefficients for the lowest bands found to be around 15000–20000 OD cm^−1^ mol^−1^ in all solvents, as shown in Figure 1A. HS‐84 is characterized by a dominating band at 414–422 nm attributed to a π‐π* excitation of the π‐conjugated thiophene rings. In HS‐167 and HS‐169 this level is split into two bands with the high‐energy transition occurring at around 360 nm, and a low‐energy band shifted to 470 and 504 nm, respectively, to be further discussed in the theoretical section. The most striking difference is that the absorbance of HS‐84 is blue‐shifted going from PBS to methanol/ethanol, whereas the low‐energy bands of both HS‐167 and HS‐169 are red‐shifted (Figure 1A). The corresponding emissions of the ligands are shown in Figure 1B. Here the spectra have been normalized to the absorbance (OD) at the excitation wavelength so that the spectral amplitude represents the quantum efficiency. Note that HS‐167 and HS‐169 in PBS are plotted with reference to the right scale‐bar being approx. 100 times smaller in magnitude and a corresponding decrease in quantum efficiency (QE). The values of absorbance and emission maxima are summarized together with other photophysical parameters in Table 1.

**Figure 2:**
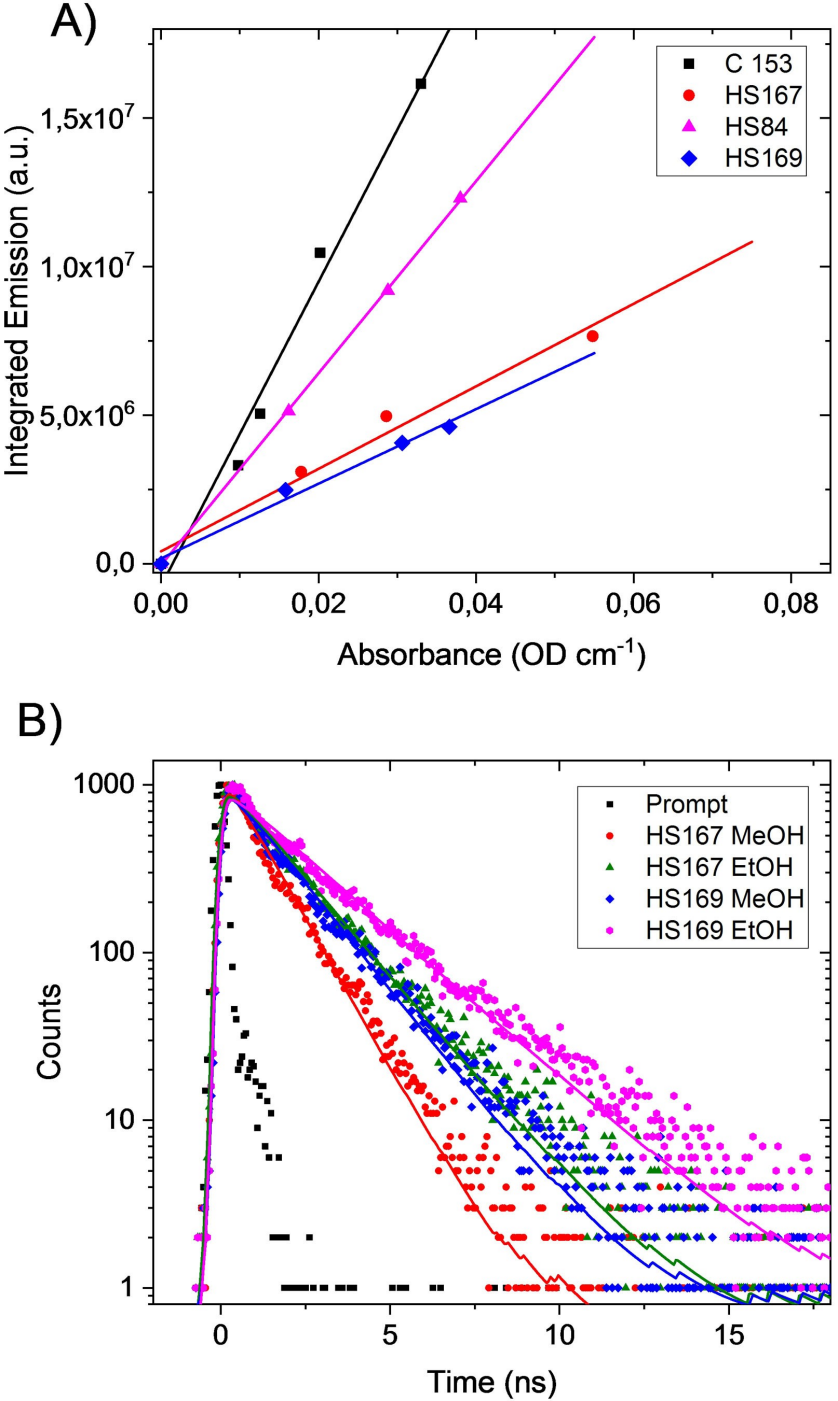
A) Slopes defined by the integrated emission vs. absorbance for determination of quantum efficiency (QE). Here methanol (MeOH) was used as solvent and Coumarin 153 (C 153) as reference (QE=0.39).^[15]^ λ_ex_=450 nm. B) Time‐correlated single photon counting (TC SPC) traces of HS‐167 and HS‐169 in MeOH and EtOH λ_ex_ : λ_em_=469 : 650 nm. Sample concentration 5 μM.

In PBS, all ligands show similar emission profiles as found previously,^[9]^ although we here pay detailed attention to the emission strength of the various bands in terms of quantum efficiency. The QE values were investigated in detail (Table 1) using the method of varying concentration and comparing with a standard reference, here Coumarin 102 and 153 in EtOH or MeOH.^[15]^ Representative slope‐data using MeOH for excitation at 450 nm are shown in Figure 2A. (The corresponding raw spectral data are shown in Figure S1 in the Supporting Information). By changing the solvent from MeOH to PBS there is a more than 50‐fold decrease in QE (Table1). Thus, both HS‐167 and HS‐169 are strongly quenched in PBS, both showing broad featureless emission bands centered at approximately 690 and 710 nm, respectively (Figure 1B). HS‐84 on the other hand, gives a partially structured emission band peaking in the 500–550 nm range, quite independent of solvent (QE changes from 26 %→30 % going from PBS to MeOH; Table 1) In addition to the low‐energy emissions, HS‐167 and especially HS‐169, also displayed an additional emission at approximately 450–500 nm when using PBS as a solvent, as displayed in Figure 1B. HS‐169 thus appear to express two distinct emission bands, which is an apparent deviation from Kasha's rule stating that fluorescence is generally occurring from the lowest excited state.^[16]^

To further investigate the quenching of the emission bands of HS‐169, samples as in Figure 1 were prepared using de‐ionized water (MQ) and deuterium oxide (D_2_O). MQ and PBS samples showed very similar absorbances and emissions, whereas the low‐energy band of the D_2_O sample was enhanced more than 4 times (Figure S2) while the high energy emission, probably resulting from S_2_ and higher electronic states, remained the same. When it comes to the isotope effect on the HS‐169 emission in H_2_O vs. D_2_O, it is very similar to findings in early work on indole derivatives, including tryptophan and tryptamine.^[17]^ In this report a concomitant increase of both the quantum efficiency and lifetime was observed, with the same order of magnitude as found here for HS‐169. This was attributed to less efficient quenching, in which C−H stretches of the excited carbons in the conjugated molecular framework couples less efficiently to the weaker OD‐stretching vibrations. The role of quantum vibrations and isotope effects was recently reviewed by Ceriotti et al.^[18]^ and their role for absorption spectra has been modelled for 9‐Methylguanine.^[19]^ Heavy water effects are also known to have impact on protein dynamics and flexibility and can then indirectly affect e. g., phosphorescence of long‐lived states.^[20]^

### 2.2 Decay Kinetics and Lifetime Imaging

The drastic changes of QE prompted us to also investigate the decay kinetics for the ligands in the different solvents. To start with PBS, MQ and D_2_O samples of HS‐169, the time‐correlated single photon counting (TC‐SPC) decay traces obtained for excitation at 469 nm and for the low energy emission are shown in Figure S2B. In MQ and PBS, the decay is very fast, on the limit of being possible to analyze using the TC‐SPC method. The fitted lifetimes were found to be equal within experimental accuracy, only around 42 ps. Using D_2_O this increased to 177 ps, which is significant and qualitatively the same increase in decay time as the amplitude of the emission found when replacing PBS/MQ with D_2_O (Figure S2). It can be concluded that the low energy emission of HS‐169 is extremely sensitive to water quenching, and it can be anticipated that vibrational properties of the solvent might play a role, here O−H have been replaced by O−D stretches by the solvent change. By changing the pH of the water solvent similar effects were noticed. At low pH, the relative contribution of high‐energy emission of HS‐169 diminished and the dominant emission around 730 nm was observed (Figure S3). This is clearly seen when normalizing the emission spectra of HS‐169 at different pH (Figure S3C). The lowering of the pH was also associated with a red‐shifted absorption (Figure S3A). HS‐167 showed a similar pH‐dependent optical characteristic as HS‐169, but the high‐energy emission band was not as pronounced as for HS‐169 (Figure S3).

**Figure 3:**
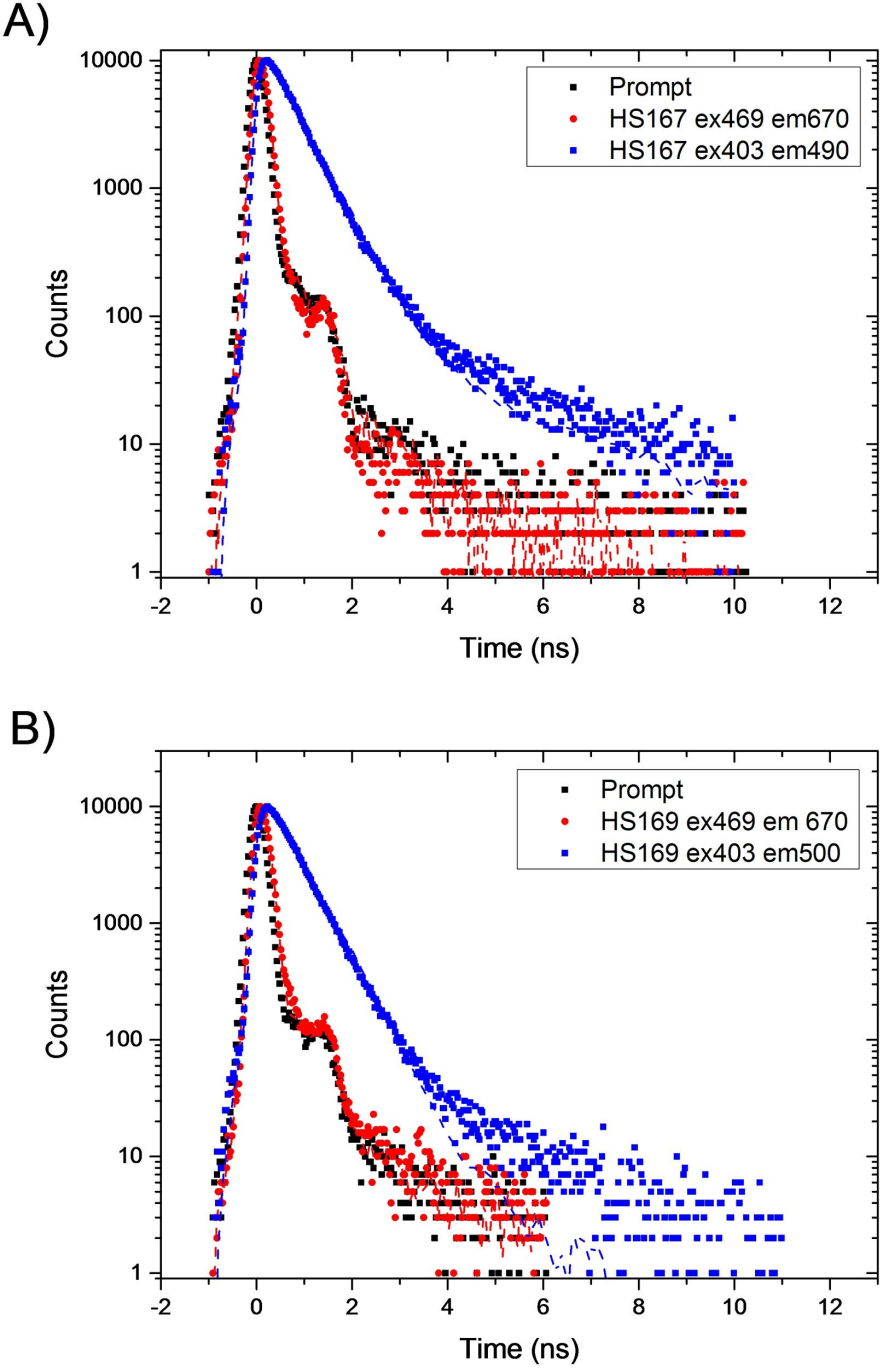
TC‐SPC traces of A) HS‐167 and B) HS‐169 in PBS. Both excited at 403 and 469 nm, and measured at the high‐energy (490 or 500 nm) and low‐energy emission bands (670 nm for both). All samples had a concentration of 5 μM. For clarity, the prompt is only shown for the 469 nm excitation.

The fluorescence lifetimes of HS‐167 and HS‐169 in PBS were measured by excitation at several different wavelengths: at 337 and 403 nm to directly excite more of the high‐energy absorption and at 469 nm to exclusively excite the low‐energy absorption (Figure 2A). The corresponding emissions were then collected at around 500 nm and 670 nm, respectively. There was a dramatic difference in the corresponding lifetimes in that the high‐energy emission displayed a longer decay time, 515 and 480 ps for HS‐167 and HS‐169, respectively, whereas the low‐energy emission was very fast, in the limit of being resolved in the range 35–45 ps (Figure 3 and S2B).

Using MeOH as solvent both the lifetimes and the QE increased dramatically for HS‐167 and HS‐169, whereas HS‐84 is comparably insensitive to these solvent effects (Figure 2; Table 1). Shifting solvent to the less polar EtOH both the QE and the decay time increased further and became comparable to decay kinetics recorded using fluorescence lifetime imaging (FLIM).

**Figure 4:**
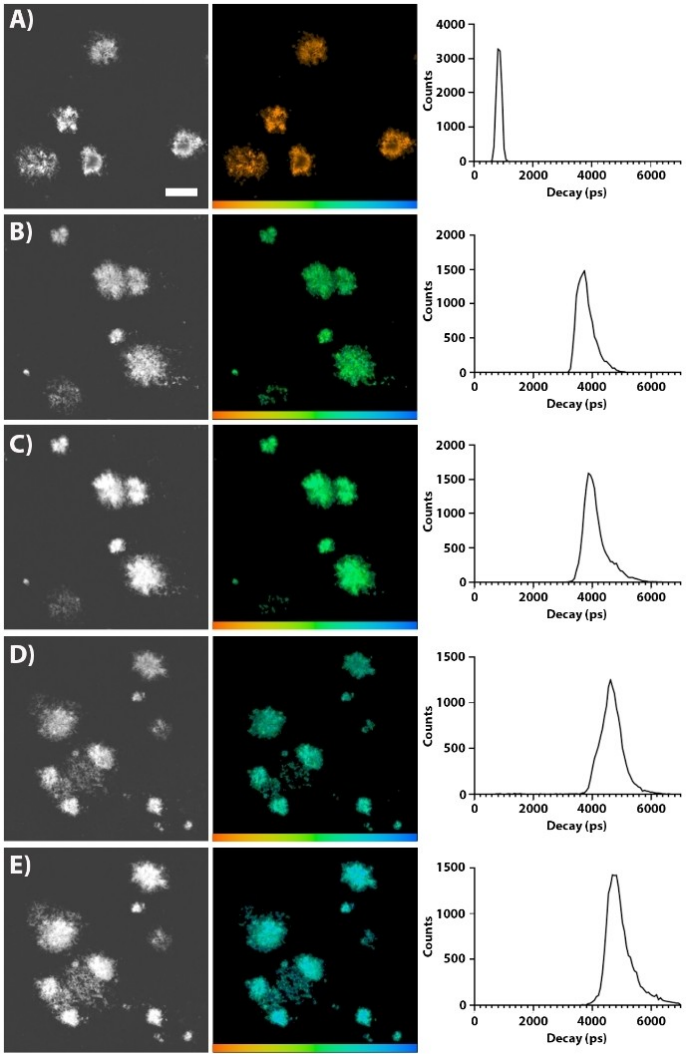
Fluorescence lifetime images and intensity‐weighted mean lifetime (t_i_) distributions of HS‐84 (A), HS‐167 (B−C) and HS‐169 (D−E) stained Aβ deposits in brain tissue section from transgenic mice (APPPS1 18 months) with AD pathology. The fluorescence lifetimes were collected with excitation at 490 nm (A, B, D) or excitation at 561 nm (C,E). The color bar represents lifetimes from 200 ps (orange) to 7000 ps (blue) and the images are color coded according to the representative lifetime. Decays were collected from 10 to 20 individual Aβ deposits. The scale bar in panel (A) represents 50 μm.

FLIM have previously been employed as a powerful technique for distinguishing polymorphic protein aggregates stained by oligothiophenes and related fluorescent ligands.^[21]^ Therefore, we next analysed the decay times of the ligands bound to Aβ deposits in brain tissue sections from APPPS1 transgenic mice with AD‐like pathology (Figure 4). The FLIM experiments were carried out with excitation at 490 nm for all three ligands and at 565 nm for HS‐167 and HS‐169. The acquired curves were fitted with a bi‐exponential decay function and two components of the fit to calculate an intensity weighted lifetime. Similar to other pentameric oligothiophenes,[^13b^, ^21a^] HS‐84 bound to Aβ deposits displayed intensity weighted lifetime (*t_i_*) distributions between 600 to 800 ps (Figure 4A), in accordance with the decay times in the solvents (Table 1), being rather insensitive to the solvent effect on QE, decay kinetics and spectral features. In contrast to HS‐84, both HS‐167 and HS‐169 showed much longer decays when bound to aggregates. HS‐167 exhibited lifetime distributions between 3 to 5 ns and similar distributions were observed when using excitation at 405 or 535 nm (Figure 4B−C). These lifetimes are strikingly longer compared to the ones obtained for HS‐167 in water (Figure 3). An analogous trend was also observed for HS‐169, since the ligand showed lifetimes ranging from 4 to 6 ns when bound to Aβ‐aggregates (Figure 4D−E). These values are of the same order of magnitude as found for HS‐167 and HS‐169 dissolved in EtOH, 1.8 and 2.4 ns, respectively (Table 1), with the decay of HS‐167 somewhat faster, just as in the FLIM case. Thus, following the trends observed for the solvent effects it can be anticipated that binding sites of the Aβ‐deposit is even more hydrophobic than the solvation cage in EtOH.

### 2.3 Two‐photon Excitation Imaging

**Figure 5:**
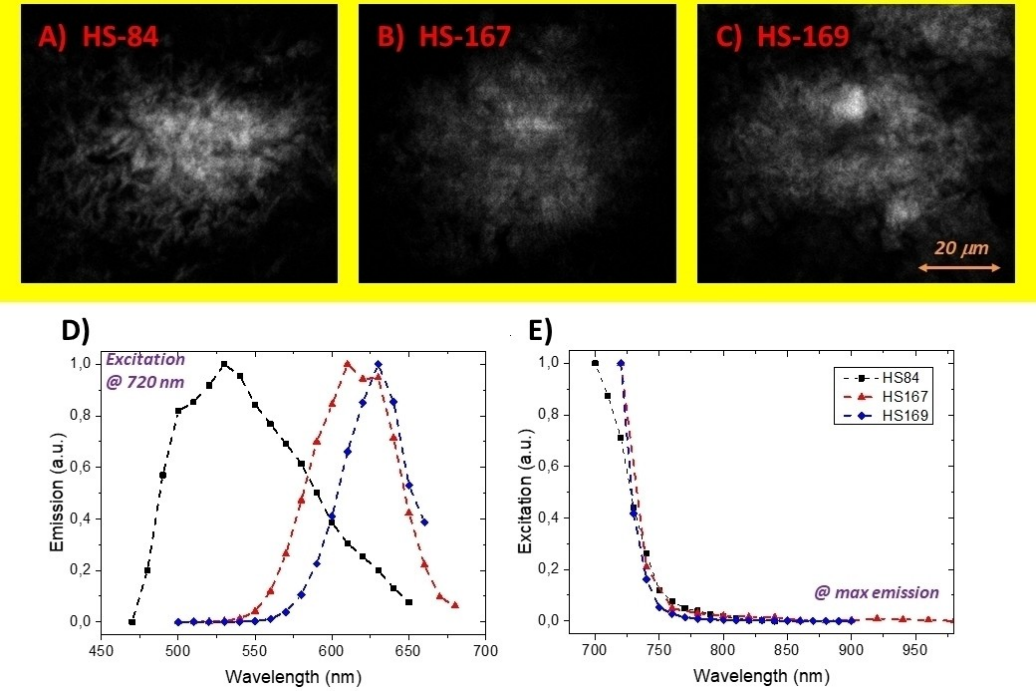
Multiphoton excitation microscopy images and spectra from extracellular Aβ deposits in brain tissue sections from transgenic APPPS1 mice (age 18 months). A−C) Images of Aβ deposits stained by the respective ligand. D−E) Emission‐ and excitation spectra of the ligands bound to Aβ deposits. HS‐84 (black square); HS‐167 (red triangle); HS‐169 (blue diamond). The dashed lines are introduced to guide the eye.

As it was recently shown that HS‐84 and HS‐169 can be utilized for longitudinal *in vivo* imaging of protein aggregates,^[10]^ we next examined the multiphoton characteristics of the ligands bound to Aβ‐deposits in brain tissue sections from APPPS1 transgenic mice with AD‐like pathology. Aβ deposits could selectively be identified by characteristic emission from the ligands (Figure 5A−C). HS‐84 displayed well‐resolved emission spectra with the characteristic double peak in the range 500–550 nm upon binding to assemblies of Aβ, whereas HS‐167 and HS‐169 showed fluorescence spectrum with red‐shifted emission between 600–650 nm (Figure 5D). Hence, all the ligands showed similar emission characteristics as previously reported for one photon excitation compared to the other ligands.^[9]^ Scanning the two‐photon excitation laser towards longer wavelengths the emission distinctly decreased above approximately 750 nm as shown in Figure 5E. This is in accordance the calculations of two‐photon cross‐section to be further discussed in the theoretical section.

### 2.4 Theoretical Investigations

**Figure 6:**
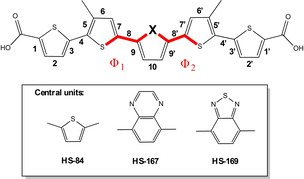
Labeling of the C−C bonds in the thiophene backbone of HS‐84, HS‐167 and HS‐169. Dihedrals Φ_1_ and Φ_2_ are marked in red.

*Conformations and bond length analysis*. The most stable conformations with respect to the dihedral angles Φ_1_ and Φ_2_ in Figure 6 were determined for each ligand. The calculations were performed on model systems of each ligand where the −CH_2_COOH groups have been replaced by −CH_3_ groups, as depicted in Figure 6. For HS‐84 and HS‐169 the *trans*/*trans* conformations were found to be the most stable, and for HS‐167 the *cis*/*cis* conformation. The terminal thiophene‐rings are all in *trans*‐conformation for all structures, as illustrated in Figure 6. In order to evaluate differences in the geometries of the ground and excited states of the three ligands, bond length alternation along the backbone of the three molecules were determined. Only the most stable conformation was selected for this analysis. Relative energies are presented in Table S1 in the Supporting Information. For HS‐84 and HS‐169 the bond distances for the *trans*/*trans* conformation and for HS‐167 the *cis*/*cis* conformation are displayed.

**Figure 7:**
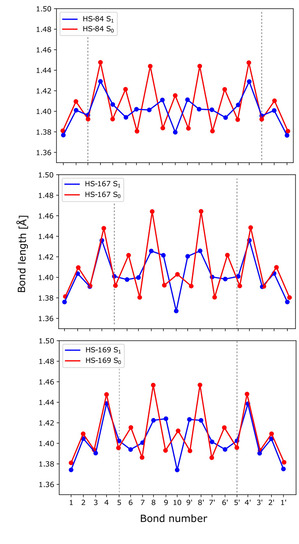
Bond length alternation pattern of the HS‐84 (*trans*/*trans*), HS‐167 (*cis*/*cis*) and HS‐169 (*trans*/*trans*) conformations in ground state (S_0_, red) and first excited singlet state (S_1_, blue). For bond labels, see Figure 6.

Previous studies of LCOs revealed that the inter‐ring bonds are shorter in the excited S_1_‐state compared to the ground state.^[22]^ Bond lengths for the three ligands were determined for the optimized ground state (S_0_, red) and the optimized first excited singlet state (S_1_, blue) for the ligands. The results are plotted in Figure 7 with the numbering of the bonds presented in Figure 6.

The results agree with our previous study of bond lengths in the ground and first excited state of other LCOs with only thiophene rings, similar to HS‐84.^[22]^ The excitation to the S_1_‐state is associated with transfer of charge from within the conjugated π‐system of the backbone of the thiophene rings to between the rings, altering the molecular structure from benzoid to quinoid. The bond length inversion that takes place during the electronic excitation from ground to the first excited state is mostly pronounced in the three inner thiophene rings. For the ligands where an acceptor group replace the central thiophene unit (HS‐167 and HS‐169) the differences in bond lengths are more pronounced in the center of the ligand, compared to the full thiophene ligand (HS‐84). For HS‐84, the bond length inversion is more evenly spread between the three inner thiophene rings. These regions are high‐lighted by the vertical dotted lines in Figure 7. The introduction of acceptor moieties in the central ring leading to a D−A−D topology is associated with a larger impact of differences in the distribution of electrons in the central region of the ligand, and less pronounced differences in bond lengths in the terminal regions, as plotted in Figure 7. Bond lengths for the S_2_ state were also determined for the optimized S_2_ state, and can be found in Figure S4. Moreover, in the S_2_ state, the bond length profiles for HS‐167 and HS‐169 are shifted compared to bond lengths of HS‐84. As could be anticipated, the largest differences in bond lengths between the S_1_ and S_2_ states were around the central unit.

**Figure 8:**
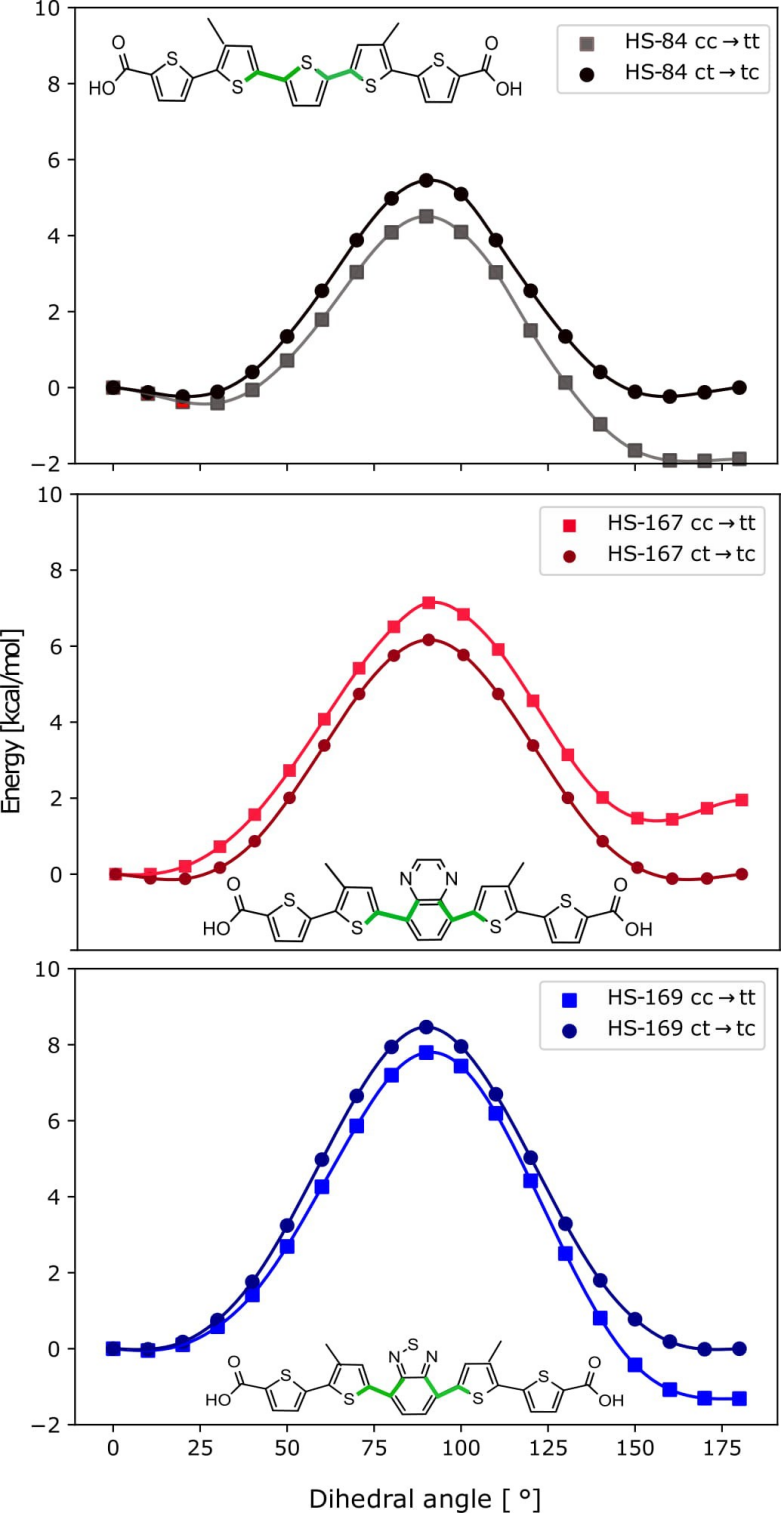
Potential energy surface scan for rotation of only the central unit compared to neighboring thiophenes, between 0–180 degrees from *cis*/*cis* (cc) to *trans*/*trans* (tt), marked by circles, and from *cis*/*trans* (ct) to *trans*‐*cis* (tc) conformation, marked by squares, for HS‐84 (black), HS‐167 (red) and HS‐169 (blue). The dihedral angles are marked in green.

*Potential energy surface scanning*. The relative differences in energy between the three optimized conformations of each ligand were smaller than 2 kcal/mol (Table S1), indicating that all three conformations are likely to exist in the experimentally studied sample. However, for the ligand to be able to explore each conformation, the rotational barrier around the central unit cannot be too high. In order to evaluate how the introduction of the more bulky central units in HS‐167 and HS‐169 affect the rotational barriers, the potential energy surface was scanned. Only the central unit was rotated with regards to dihedral angles to neighboring thiophene rings between 0–180 degrees, the rest of the molecule was still in one plane. The dihedral angles are defined as Φ_1_ and Φ_2_ in Figure 6, and marked in green in Figure 8. The results of the dihedral scan originating in *cis*/*cis* conformation, ending up in *trans*/*trans* are depicted as squares in Figure 8 for HS‐84 in black, HS‐167 in red and HS‐169 in blue. The scan originating in the *cis*/*trans*, ending up in the *trans*‐*cis* conformation are plotted as circles in the same figure.

As expected, the rotational barriers were smallest for HS‐84, which has the smallest central unit of the three ligands. The rotational barrier is relatively low for HS‐84 going between the most stable *trans*‐*trans* conformations to a *cis*‐*cis* conformation, 6.4 kcal/mol, plotted as black squares in Figure 8. The barrier going back from the less favorable *cis*‐*cis* conformation to *trans*‐*trans* is 4.5 kcal/mol. Starting in a *cis*‐*trans* conformation, and going to a *trans*‐*cis* conformation requires 5.8 kcal/mol, plotted in black circles in Figure 8. For comparison, the rotational barrier for a completely unsubstituted bithiophene is 1.5 kcal/mol. The barrier for HS‐167 going from the most stable conformation, *cis*‐*cis* to *trans*‐*trans* is 7.2 kcal/mol (and 6.3 from *cis*‐*cis* to *trans*‐*trans*, red squares), and 6.5 kcal/mol (red circles) from *cis*‐*trans* to *trans*‐*cis*. The larger barriers of HS‐167 and HS‐169 means that these variants will be less prone to twist around these dihedral angles, and will thus spend more time in a planar conformation. Since the planarity of anionic thiophene molecules have been found to be strongly correlated to shifts in transition wavelengths,[^14^, ^18^] the differences in rotational barriers can help to explain the experimentally observed red‐shift of HS‐167 and HS‐169 compared to HS‐84.

To conclude this section, the introduction of the QX and BTD moieties contributes to an increased stiffness of the ligands. Increased rigidity implies that the molecules will spend more time in a planar conformation since fewer rotations will occur. In turn, this leads to that the π‐orbitals in the thiophene backbone will be overlapping more often, and thus form a more efficient π‐system. Shifts in transition wavelengths of about 100 nm have previously been ascribed to changes in planarity of anionic thiophenes.^[11]^

The central QX unit in HS‐167 contains two additional conjugated bonds compared to HS‐84, making the conjugated system larger in HS‐167 than in HS‐84. Rotation of the central unit breaks the conjugation, which is associated with a larger penalty for a larger conjugated system. Furthermore, when HS‐167 is in a planar conformation, there are stabilizing hydrogen bonds between a sulphur atom and hydrogen on the central unit, as well as a stabilizing interaction between nitrogen and sulphur (depicted in Figure S5), that is lost when the central unit is rotated. Finally, the largest rotational barrier was identified for HS‐169 at 9.3 kcal/mol from the most stable *trans*‐*trans* conformation (8.0 kcal/mol from *trans*‐*trans* to *cis*‐*cis*) plotted as blue squares. The rotational barrier from *cis*‐*trans* to *trans*‐*cis* was 8.8 kcal/mol, plotted as blue circles. In HS‐169 there is a favorable interaction between the nitrogen atoms in the central unit and the sulphur atoms in the thiophene groups (depicted in Figure S5), which contributes to the larger barrier for this ligand, since these favorable interactions are lost when the central unit is rotated. This larger rotational barrier can also be attributed to that the larger conjugated system, compared to HS‐84, is broken.

*Absorption spectrum calculations*. The absorption spectrum for the transition between S_0_ and S_1_ for the *trans*/*trans*, *cis*/*trans* and *cis*/*cis* conformations were calculated for the ligands in order to determine how the absorption spectra are affected by the conformations of the ligands.

**Figure 9:**
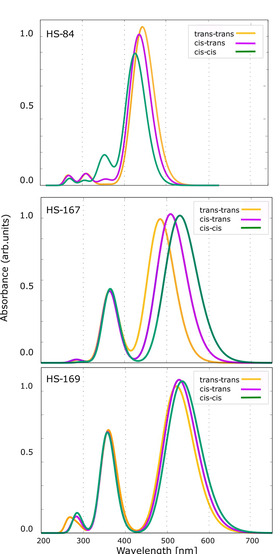
Theoretical absorption spectra for different conformations (*cis*‐*cis* in green, *cis*‐*trans* in purple and *trans*‐*trans* in yellow) of HS‐84 (top), HS‐167 (middle) and HS‐169 (bottom).

In both the experimentally measured absorption spectra shown in Figure 1A as well as in the calculated spectra presented in Figure 9, HS‐84 displays a single strong absorption peak around 450 nm. The *cis*‐*cis* conformation is colored in green, the *cis*‐*trans* conformation in purple and *trans*‐*trans* in yellow. HS‐167 and HS‐169 both display strong peaks in the region of 500–550 nm corresponding to experimentally observed peaks in this region. HS‐167 and HS‐169 also display second peaks in a higher energy range at around 350 nm. The peaks in the higher energy region have been attributed to arise due to charge‐transfer within the D−A−D system that is introduced by the change of central unit in HS‐167 and HS‐169.^[9]^ The most dominant peak is attributed to π‐π* transitions in the conjugated thiophene backbone of all three ligands. The different conformations of HS‐167 display the largest relative shift in absorption peaks, of about 70 nm. In contrast, the excitation energies of the different conformations of HS‐84 (top) and HS‐169 (bottom) are in a very similar wavelength range (of about 10–20 nm). The results are summarized in Table S2. While the main calculated absorption peak for HS‐84 is around 450 nm, it is shifted to around 500–530 nm for HS‐167 and HS‐169. This drastic shift in transition wavelength is also observed in the experimental spectra of the ligand in solution in Figure 1A. The experimentally observed difference in transition wavelength is about 150 nm, and thus even more pronounced than the calculated ones. We attribute this discrepancy to that in the experimental sample there are even more conformational variations than the three included in the calculations. In order to obtain a more representative sample it would be necessary to conduct molecular dynamic simulations to fully sample the conformation space, which is computationally demanding and out of the scope of this study. Another factor, which could alter the computational result, is to include environment models for the response calculations.

The experimentally detected multiphoton‐acquired shift in emission of the bound ligands in Figure 5D is around 100 nm, and thus less pronounced than the shift observed for the ligands in solution. When the ligands are bound to the amyloid fibrils, as the scaffold of the binding pocket will restrain their movements, thus leading to smaller variations in conformation of all bound ligands, which is reflected in the smaller shift in transition wavelengths of the bound probes.

Table S2 summarizes numerical values of excitation energies and oscillator strengths for the transition between S_0_ and S_1_ for the three investigated conformations for each of the three ligands. The differences in transition wavelengths between the three conformations of HS‐84 are 19.1 nm, for HS‐167 it is 42.6 nm, and for HS‐169 it is 13.4 nm. Even though the differences in transition energies for the different conformations are larger for HS‐167 compared to the other two ligands, the different conformations of the three ligands do not give rise to distinctly different spectral profiles.

*Fluorescence spectrum calculations*. The experimentally obtained fluorescence spectra of the three ligands in water and methanol are presented in Figure 1B. HS‐84 display one distinct peak around 530 nm, while HS‐167 and HS‐169 both display one dominant peak around 630–650 nm, as well as a less intense peak around 450 nm in PBS. The theoretical results of the transition between S_1_ and S_0_ are summarized in Table 2. All three ligands show a similar trend between the different conformations, where the largest transition energy is found for the *trans*/*trans* conformation for HS‐84, HS‐167 and HS‐169, as presented in Table S2 in the SI. However, comparing the experimentally obtained fluorescence spectra with the calculated transition energies, it is clear that the total experimental spectral profiles contain contributions that are not represented by transitions between the S_1_ state and the ground state, S_0_. All calculated transitions between S_1_ and S_0_ take place within a wavelength range of about 100 nm. Based on this, it can be concluded that the peaks in the higher energy‐region for HS‐167 and HS‐169 compared to HS‐84 cannot be attributed to arise due to different conformations of each ligand. Therefore, we also performed calculations of the transitions between S_2_ and S_0_. These transitions occur with a blue‐shift of about 200 nm compared to the S_1_ to S_0_ transitions, and are therefore the likely source of the fluorescence signals in the shorter wavelength range in Figure 1B. The numerical results of transitions between S_2_ and S_0_ are summarized in Table 3.

**Table 2:** Vertical transition energies and oscillator strengths for the S_1_ to S_0_ transition. Relative transition energies are given with respect to the conformation with lowest S_1_‐state energy.

Ligand	Conf.	S1‐S0 (E) [eV]	S1‐S0 (λ) [nm]	ΔE [eV]	Δλ [nm]	osc.
**HS‐84**	*trans*/*trans*	2.31	535.8	0.04	−11.0	1.89
	*cis*/*trans*	2.28	543.1	0.01	−3.7	1.81
	*cis*/*cis*	2.27	546.8	0.00	0.0	1.61
**HS‐167**	*trans*/*trans*	2.17	570.2	0.08	−22.6	1.26
	*cis*/*trans*	2.13	582.0	0.04	−10.8	1.29
	*cis*/*cis*	2.09	592.8	0.00	0.0	1.26
**HS‐169**	*trans*/*trans*	2.02	614.0	0.06	−18.6	1.13
	*cis*/*trans*	1.99	623.6	0.03	−9.0	1.17
	*cis*/*cis*	1.96	632.6	0.00	0.0	1.15

**Table 3:** Vertical transition energies and oscillator strengths for the S_2_ to S_0_ transition. Relative transition energies are given with respect to the conformation with lowest S_2_‐state energy.

Ligand	Conf.	S2‐S0 (E) [eV]	S2‐S0 (λ) [nm]	ΔE [eV]	Δλ [nm]	osc.
**HS‐84**	*trans*/*trans*	3.32	373.0	0.00	0.0	0.01
	*cis*/*trans*	3.33	371.7	0.01	−1.3	0.08
	*cis*/*cis*	3.36	368.7	0.04	−4.3	0.41
**HS‐167**	*trans*/*trans*	3.29	377.7	0.08	−7.9	0.29
	*cis*/*trans*	3.25	381.8	0.04	−3.8	0.06
	*cis*/*cis*	3.21	385.6	0.00	0.0	0.02
**HS‐169**	*trans*/*trans*	3.21	386.2	0.09	−10.8	0.17
	*cis*/*trans*	3.17	391.5	0.05	−5.5	0.03
	*cis*/*cis*	3.12	397.0	0.00	0.0	0.02

The oscillator strengths for some of the S_2_ to S_0_ transitions are very low (summarized in Table 3), leading us to suspect that the potential energy surfaces of S_2_ and S_3_ states are energetically close, and that the measured fluorescence signal might be a mixed band from both S_2_ and S_3_ states to the ground state S_0_. After performing optimizations also of the S_3_ state, it could be concluded that the two additional peaks identified for HS‐169, and lesser extent HS‐167, are most likely due to transitions from a combination of S_2_ and S_3_ states to S_0_ (Tables 2–4). As presented in Table 4, HS‐84 also exhibits transitions from the S_2_ and S_3_ states to S_0_ with a blue‐shift of around 70 nm. The experimental detection range is only down to 400 nm, so this less intense peak of HS‐84 is likely just out of the visible wavelength range.

**Table 4:** Vertical transition energies and oscillator strengths for the S_3_ to S_0_ transition. Relative transition energies are given with respect to the conformation with lowest S_3_‐state energy.

Ligand	Conf.	S3‐S0 (E) [eV]	S2‐S0 (λ) [nm]	ΔE [eV]	Δλ [nm]	osc.
**HS‐84**	*trans*/*trans*	3.56	347.8	0.02	−2.4	0.00
	*cis*/*trans*	3.55	348.9	0.01	−1.3	0.00
	*cis*/*cis*	3.54	350.2	0.00	0.0	0.00
**HS‐167**	*trans*/*trans*	3.28	377.7	0.00	0.0	0.28
	*cis*/*trans*	3.31	374.4	0.03	−3.3	0.78
	*cis*/*cis*	3.32	373.4	0.04	−4.3	0.82
**HS‐169**	*trans*/*trans*	3.24	382.6	0.00	0.0	0.80
	*cis*/*trans*	3.25	381.1	0.01	−1.5	0.87
	*cis*/*cis*	3.27	379.0	0.03	−3.6	0.89

The separation between the two peaks in the experimentally determined fluorescence spectrum is about 200 nm, which agrees well with the relative distance between the theoretically obtained transitions. However, the absolute values of the calculated transitions are heavily dependent on the choice of functional, and the extent of Hartree–Fock‐exchange that is included in the method. In this work, the Coulomb attenuated method CAM−B3LYP^23^ exchange correlation functional was employed, as it has previously been shown to be suitable for calculations of ground and excited states of LCOs.[^9^, ^22^, ^23^] For these calculations the functional did not fully capture the specific values of the transitions, but the relative differences in absorption peaks agree well with experimental data. Nevertheless, due to the introduction of the D−A−D moiety, both HS‐167 and HS‐169 display a striking red‐shift of the fluorescence profile compared to HS‐84, as well as second peaks in the high‐energy region about 200 nm lower than the dominant peaks in both experimental measurements and theoretical results. We propose that this is due to the increased quinoid character of HS‐167 and HS‐169 that is associated with a higher degree of conjugation in the π‐system in the thiophene rings.

**Figure 10:**
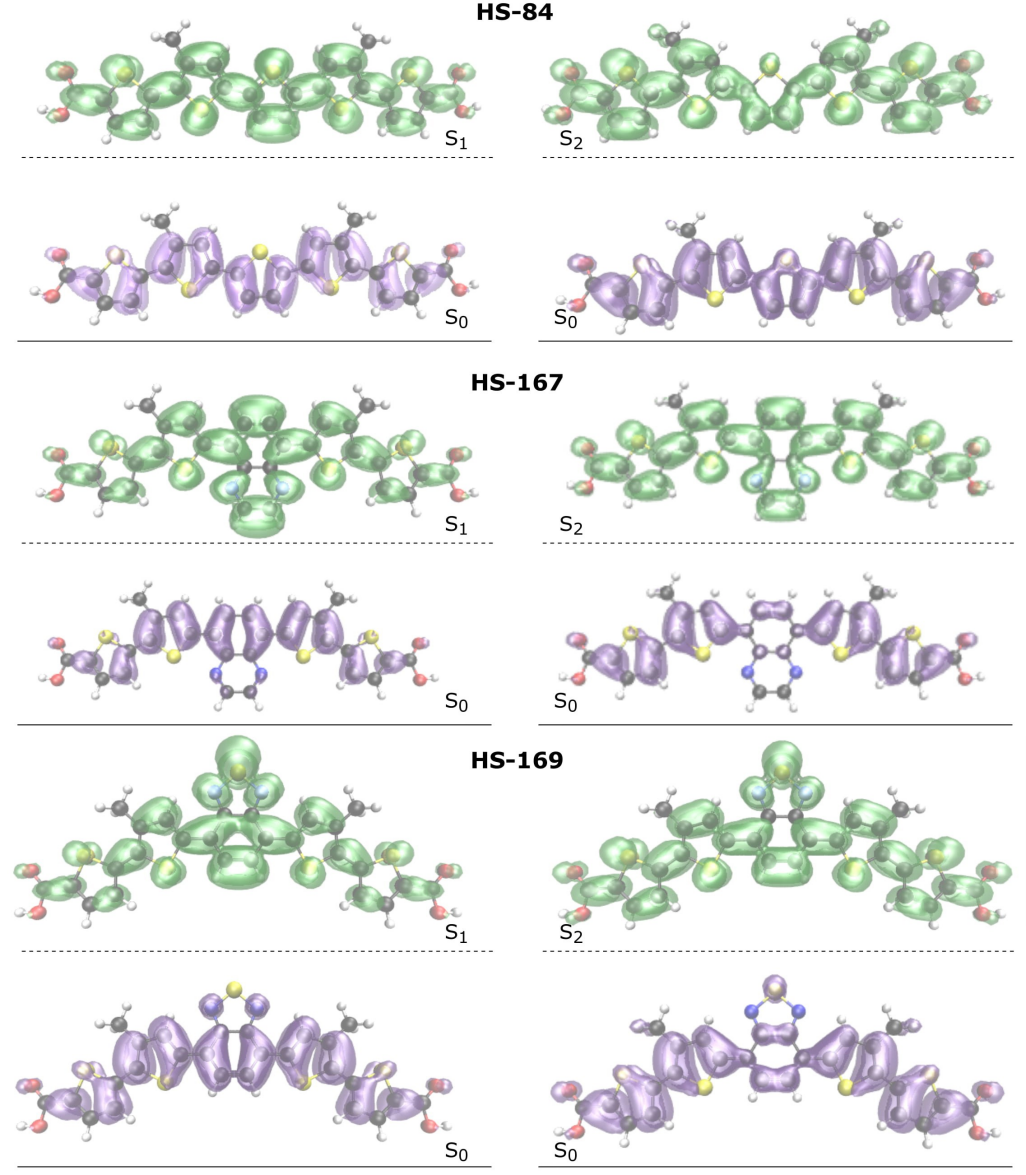
Detachment (purple) and attachment (green) densities for the transition between S_1_ and S_0_, and S_2_ and S_0_ for the most stable conformation of each ligand.

*Detachment and attachment densities*. In order to study the excited states S_1_ and S_2_ in more detail, detachment and attachment densities were calculated for the most stable conformation of each ligand. The detachment densities display from which regions electron density is removed from the ground state during the excitation process. Attachment densities display where the electron density has been added in the excited state.^[20]^ When an electronic transition takes place, all orbitals will be more or less affected by the re‐distribution of electron density in the molecule. These densities reveal more than the traditional analysis of identifying which molecular orbitals are involved in an electronic transition since the orbitals are allowed to relax due to the shift in electron density that takes place during the electronic transition.^[25]^ This effect is very pronounced when non‐valence electron transitions are considered, since core‐excitations are associated with a larger degree of orbital relaxation compared to valence excitations. Figure 10 displays the detachment (purple) and attachment (green) densities for the first excited singlet state S_1_, and second excited singlet state S_2_. As expected, for all three ligands, electron density is removed from the conjugated π‐system in the thiophene rings, and re‐distributed to the inter‐ring C−C bonds, as visualized in Figure 10. The re‐distribution of electron density is more uniformly shifted along the thiophene rings for the different electronic states of HS‐84. A larger degree of charge‐transfer character can be observed for the extension of the HS‐167 and HS‐169 molecules. In the S_1_ state the electron density is dominantly added to the BTD and QX groups, as well as the bonds to the neighboring thiophene units. In the S_2_ state the electron density is foremost shifted to the terminal regions of the two ligands. This can be compared to the bond length analysis, where differences in bond lengths are more evenly spread over the three inner thiophene rings for HS‐84, while more localized on the central group and bonds to the neighboring rings for HS‐167 and HS‐169, as plotted in Figure 7 and Figure S6 in the SI.

Larger electron densities between nuclei are associated with shorter bond distances between those nuclei,^[26]^ and a more pronounced quinoid character is associated with a more efficient conjugation, and this can be observed in the re‐distribution of electron density for the dominant S_0_ to S_1_ transition. We propose that these differences in electron densities in the excited state adds to the result that HS‐167 and HS‐169 are less flexible (also in the excited state) due to the introduction of the BTD and QX groups. This increased rigidity promotes orbital overlap of the π‐system, and this will influence the spectroscopic profile of the dominant π‐π* transition of the ligands. Difference densities between detachment and attachment of the ground and two lowest electronic excited states are visualized for additional representation of the variation of re‐distribution of electron densities for the different electronic states, and can be found in Figure S4.

**Figure 11:**
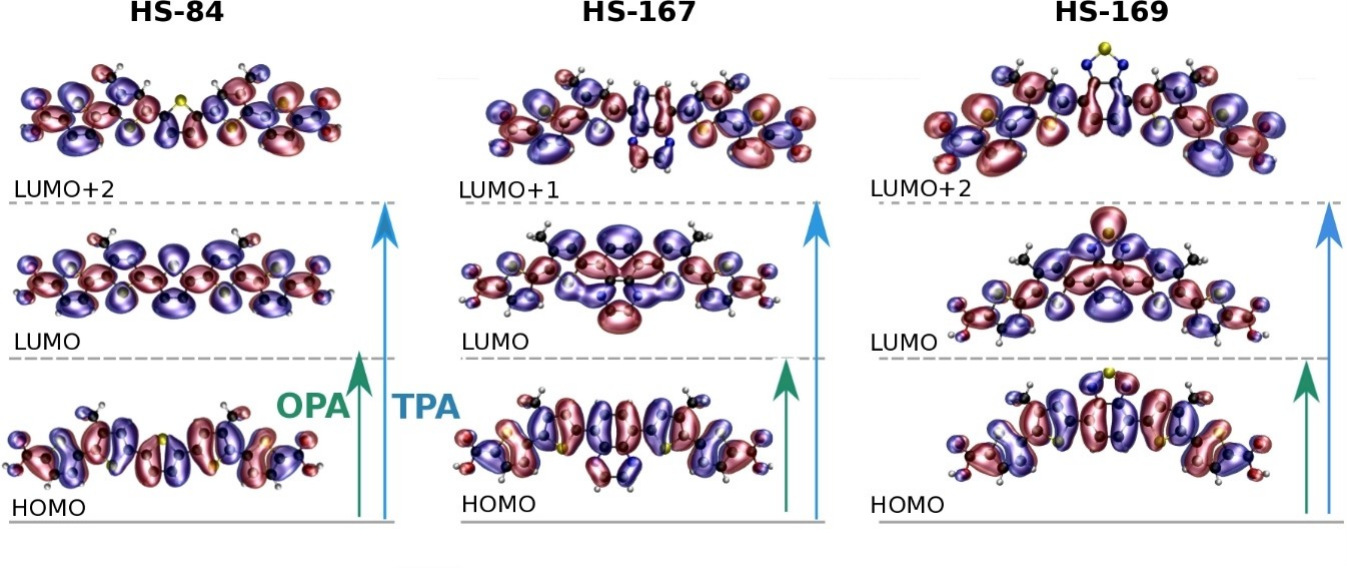
The dominant contributions of the OPA are from electronic transitions between HOMO and LUMO in the most stable conformation for each ligand (green arrows). The dominant contributions of the TPA signal are between HOMO and LUMO+2 for HS‐84, LUMO+1 for HS‐167 and LUMO+2 for HS‐169 (blue arrows).

*TPA calculations*. When studying molecules with OPA and TPA, different electronic states are probed, and there are different selection rules for the two. Thus, comparing how the TPA spectra vary depending on the molecular conformation for the three ligands can give additional information about the systems. Figure 11 displays the orbitals involved in the dominant electronic transitions for the most stable conformation of each ligand. This representation visualizes the extent of orbitals of the different electronic states that are probed with OPA and TPA. In the highest occupied molecular orbital (HOMO), the electron density is mainly located within the conjugated backbone of the thiophene rings. In the lowest unoccupied molecular orbital (LUMO) the electron density is shifted to the inter C−C bonds.

The orbitals that are probed with TPA have the same symmetry as the HOMO, while the orbitals involved in the HOMO‐LUMO transition have different symmetries. The LUMO orbitals are symmetric, while the other two are non‐symmetric. For the different conformations of HS‐84 and HS‐169, the difference in energies of the two‐photon allowed states changes is small (corresponding to 12.4 nm and 4.0 nm, respectively). For HS‐167, just as the energy of the S_1_ state, the energy changes significantly between the conformations of HS‐167 (25.2 nm). Table 5 summarizes the calculated TPA results, and by comparing with the experimental TPA spectra in Figure 5E it is evident that the stronger TPA transitions are outside the available excitation region towards the short wavelength (high‐energy) side. Thus, the strongest experimental emission spectra are obtained by exciting 720 nm (Figure 5D), while the calculations reveal that the majority of the strong two‐photon bands fall within the range of 598–676 nm. Unfortunately, this also overlaps with the emission spectra. Therefore, it would be desirable to synthesize ligands with large TPA cross sections at a longer wavelength range in order to increase and optimize the TPA induced emission signal.

**Table 5:** Photon energies and wavelengths and TPA cross‐sections for the strongest two‐photon bands in the visible region.

Ligand	Conf.	E [eV]	λ [nm]	σ_TPA_ [a.u.]
**HS‐84**	*trans*/*trans*	2.03	610.8	1.63*10^4^
	*cis*/*trans*	2.05	604.2	1.45*10^4^
	*cis*/*cis*	2.07	598.4	1.34*10^4^
**HS‐167**	*trans*/*trans*	1.95	636.4	1.22*10^4^
	*cis*/*trans*	1.91	649.0	1.39*10^4^
	*cis*/*cis*	1.88	659.6	1.35*10^4^
**HS‐169**	*trans*/*trans*	2.33	671.6	3.87*10^5^
	*cis*/*trans*	1.84	673.6	1.52*10^4^
	*cis*/*cis*	2.00	675.6	1.34*10^4^

## 3 Conclusions

The introduction of central units with D−A−D properties into a pentameric thiophene oligomer leads to an extended range of possible spectroscopic detection of different morphologies of disease‐associated proteins, without reducing the selectivity of the ligands. It was shown experimentally how the introduction of the substituents made the ligands much more exposed to quenching from a water based solvent, a phenomenon that explains the dramatic increase in apparent quantum efficiency when binding to hydrophobic sites in protein aggregates and related biomolecular complexes. The dramatic shift in transition energies associated with the change in the central unit is mainly due to an increased efficiency of the conjugation of the thiophene backbone for HS‐167 and HS‐169 due to favorable interactions between the central ring and neighboring thiophene rings. Different rotational conformations of the three ligands are relatively close in energy and the ligands can show dramatic differences in spectral signatures due to the complicated interplay between the excited S_1_, S_2_ and S_3_ states. Taken together, the impact of the excited states on changes in lifetimes and spectral features give a wide range of probing potential using the D−A−D enhanced ligands in advanced fluorescence microscopy based on FLIM and TPA‐excitation. The results guide the further development of fluorescence biosensing ligands and also lay foundations for modelling of more detailed structure‐to‐property relationships based on molecular dynamics simulations of the fluorescent ligands interacting with various biomolecular complexes.

## Experimental Section

### Basic Optical Spectroscopy

Steady state absorption spectra were recorded using a Shimadzu UV‐1601PC spectrophotometer. Measurements were performed with 10 mm quartz cuvettes (Hellma Precision). Steady state photoluminescence measurements were carried out employing a PTI Quantamaster 8075–22 (Horiba Scientific) equipped with Double Mono 300 spectrometer chambers for both excitation and emission. A Hamamatsu R928 PMT was used for detection in the range 185–950 nm. As light source the OB‐75X (75 W Xenon arc lamp) was used. Data acquisition and basic data‐handling of steady state luminescence data were carried out with the Felix Data Analysis software and further processed and presented using Origin Pro. Time‐resolved fluorescence decays were recorded using an IBH time‐correlated single photon counting (TCSPC) spectrometer system with 1 nm resolved emission monochromator (5000 M, Glaskow, UK). The system was equipped with a TBX‐04D picosecond photon detection module and the sample was excited using an IBH LED operating at 337, 403 and 469 nm. The measured decay‐trace was analyzed using deconvolution fitting with the IBH Data Station v 2.1 software and presented using the Origin Pro software.

#### Characterization of HS‐167 and HS‐169 at Different pH

HS‐167 and HS‐169 stock solutions (1.5 mM ligand in de‐ionized water) were diluted in 20 mM Na‐citrate buffer pH 3.0, 20 mM Na‐acetate buffer pH 4.0 or pH 5.0, and 20 mM Na‐phosphate buffer pH 6.0 or pH 7.0 to a final concentration of 3 μM. Emission spectra were recorded with an Infinite M1000 Pro microplate reader (Tecan, Männedorf, Switzerland). Data from triplicate experiments were processed and analyzed using Prism 6 (Graphpad, USA).

#### Staining of Brain Tissue Sections

Frozen brain sections (20 μm) from brain tissues from APPPS1 transgenic mice (18 months) were fixed in 96 % EtOH, rehydrated in 50 % EtOH followed by de‐ionized and then incubated in phosphate buffered saline (PBS, 10 mM phosphate, 140 mM NaCl, 2.7 mM KCl, pH 7.4) for 10 min. HS‐84, HS‐167 or HS‐169 were diluted to 600 nM in PBS and added to the sections. After 30 min, the sections were washed with PBS and mounted with Dako fluorescent mounting medium (Dako Cytomation, Glostrup, Denmark). The mounting medium was allowed to solidify overnight before evaluated with OPA and TPA excitation confocal imaging or FLIM. The brain tissue sections with AD like pathology was provided by Prof. Frank Heppner, Department of Neuropathology, Charité‐Universitätsmedizin Berlin, Germany.

#### FLIM

Fluorescence lifetime images were acquired using an inverted Zeiss (Axio Observer.Z1) LSM 780 microscope equipped with a 32 channel QUASAR GaAsP spectral array detector. In this setup the emitted photons were routed through the direct coupling (DC) confocal port of the Zeiss LSM 780 scanning unit and detected by a Becker & Hickl HPM‐100‐40 hybrid photomultiplier tube (PMT). The data were recorded by a Becker & Hickl Simple‐Tau 152 system (SPC‐150 TCSPC FLIM module) with the instrument recording software SPCM version 9.42 in the FIFO image mode using 256 time channels (Becker & Hickl GmbH, Berlin, Germany). For all acquisitions a Plan‐Apochromat 40×/1.3 Oil DIC objective lens was used, and the pinhole was set to 20.2 μm. For excitation at 405 nm a Laser diode 405 nm CW/PS with a repetition rate of 50 were utilized, whereas a pulsed tunable In Tune laser with a repetition rate of 40 MHz were used for excitation at 565 nm. Data was analyzed in SPCImage version 3.9.4 (Becker & Hickl GmbH, Berlin, Germany). Typically, decays fitted to a bi‐exponential decay and the associated life‐times and weights were used to calculate an intensity averaged life‐time for plots and comparison.^[27]^

#### Two‐photon Excitation and Emission Imaging

Amyloid structures of stained sections were visualized using a Leica SP8 SMD/MP microscope equipped with a Coherent Chameleon laser for multiphoton excitation. A 25x HCX IR Apo objective was used and images were collected using the internal PMT‐detectors for spectral detection.

#### Molecular Structure Optimizations

For each of the three ligands (Figure 1), three different conformations have been studied with different relative orientations of the central unit relative the inner thiophene rings, Figure 6. All calculations have been performed with model systems where the inner carboxyl groups have been replaced with methyl groups. The terminal thiophene rings are all in *trans*‐conformation for all structures. The different conformations are referred to with the relative conformation of the inner rings, i. e. *trans*/*trans*, *cis*/*trans* and *cis*/*cis*. All ground state (S_0_) equilibrium structures have been optimized at the B3LYP/6‐31+G(d) level of theory. The S_1_, S_2_ and S_3_ excited state structures have been optimized at the level of CAM−B3LYP/6‐31+G(d). These calculations were performed with use of the Gaussian program^[30]^

#### Absorption and Fluorescence Calculations

For TD‐DFT vertical excitation energy calculations at the respective equilibrium structures, the aug‐cc‐pVDZ^[28]^ basis set was used in combination with the long‐range‐corrected functional CAM−B3LYP^[23]^ for an adequate description of the charge‐transfer character of the states present in the D−A‐D systems. The calculations were performed with use of the Gaussian program. Two‐photon absorption were calculated using same basis set and level of theory, with the use of the Dalton program^31^ using the same procedure as described in our previous paper.^[32]^

**Attachment and detachment electron densities**. This information can be used in order to further analyze the vertical electronic excitations between the ground state S_0_ and different electronic excited states. The detachment density is determined by removing ground state density corresponding to one electron.^[24]^ The removal affects the other electrons, and a re‐distribution of the ground state density is allowed to compensate for the removal of electron density. Then electron density is then added to an excited state of the system with the newly re‐distributed electrons. These calculations were performed with CAM−B3LYP/aug‐cc‐pVDZ using the QChem software.^[29]^ By plotting the eigenvalues of the difference density matrix of the natural difference orbitals it is possible to visualize the attachment and detachment densities.

## Conflict of interest

The authors declare no conflict of interest.

## Supporting information

As a service to our authors and readers, this journal provides supporting information supplied by the authors. Such materials are peer reviewed and may be re‐organized for online delivery, but are not copy‐edited or typeset. Technical support issues arising from supporting information (other than missing files) should be addressed to the authors.

Supplementary
