# Supplementary material for: Deciphering the Electronic Transitions of Thiophene‐Based Donor‐Acceptor‐Donor Pentameric Ligands Utilized for Multimodal Fluorescence Microscopy of Protein Aggregates
Source: Chemphyschem. 2020 Dec 23;22(3):323–35. doi: 10.1002/cphc.202000669 (PMC7898931; doi:10.1002/cphc.202000669)
Supplement: Supplementary file 1 — Supplementary [file CPHC-22-323-s001.pdf]

ChemPhysChem

Supporting Information

Deciphering the Electronic Transitions of Thiophene-Based Donor-Acceptor-Donor Pentameric Ligands Utilized for Multimodal Fluorescence Microscopy of Protein Aggregates

Camilla Gustafsson, Hamid Shirani, Petter Leira, Dirk R. Rehn, Mathieu Linares, K. Peter R. Nilsson, Patrick Norman, and Mikael Lindgren*

Supporting Information

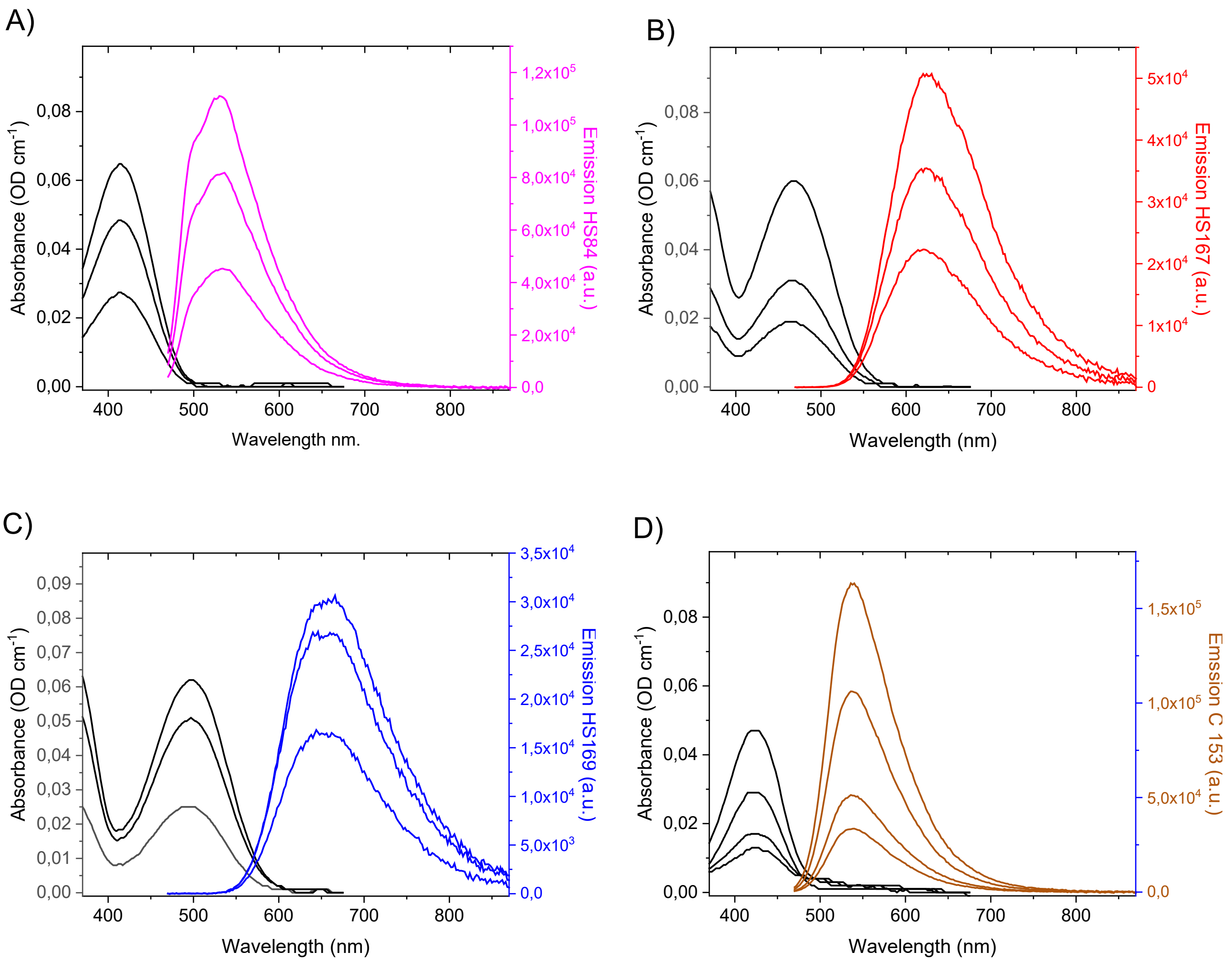

Figure S1. Raw spectral data for QE slope calculations (Figure 2A). A) HS84; B) HS167; C) HS169; D) Reference Coumarin 153 (C 153). Solvent: MeOH for all cases, λ_{ex} = 450 nm for the emission spectra.

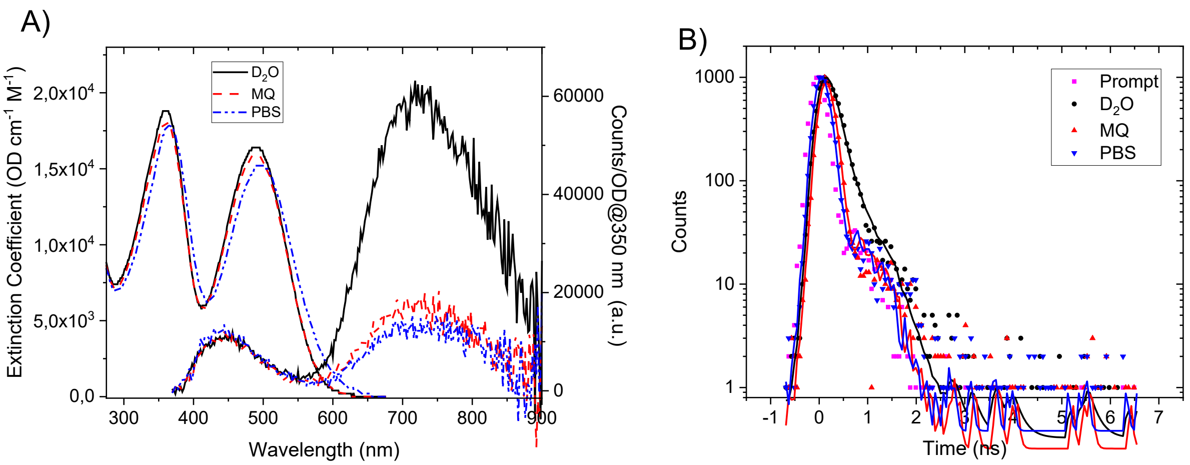

Figure S2. A) Absorbance and emission spectra of HS169 in PBS, D_2O and de-ionized water (MQ). (**B**) TC-SPC traces of the samples in A). λ_{ex} = 469 nm; λ_{em} = 650 nm. The solid lines are fits to a mono-exponential with the following decay times, PBS: 44 ± 4 ps, MQ: 40 ± 7 ps, D_2O: 177 ± 4 ps.

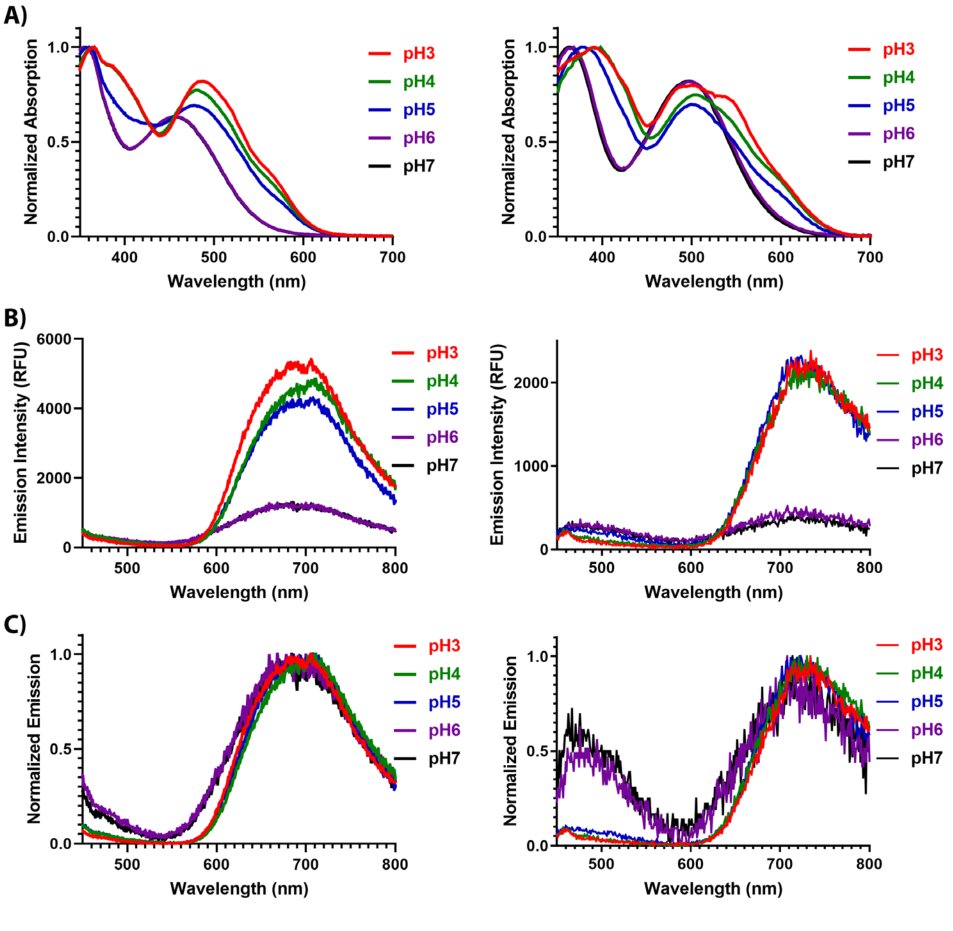

Figure S3. pH-dependent chromic transitions of HS-167 and HS-169. Normalized absorption- (**A**), Emission- (**B**) and normalized emission spectra (**C**) of HS-167 (left panel) and HS-169 (right panel) at different pH. Excitation at 360 (HS-167) and 380 nm (HS-169).

Table S1: Conformations, dihedral angles (marked in red in Figure 6) and relative ground state energies for the different conformations of HS-84, HS-167 and HS-169.

Ligand	Conformation	Φ1	Φ2	ΔE_0 [kcal/mol]
**HS-84**	*trans*/*trans*	180.0	180.0	0.00
	cis/*trans*	24.7	164.0	0.94
	cis/*cis*	26.3	26.3	1.88
**HS-167**	*trans*/*trans*	155.5	155.5	0.91
	cis/*trans*	2.5	157.2	1.95
	cis/*cis*	0.0	0.0	0.00
**HS-169**	*trans*/*trans*	180.0	180.0	0.00
	cis/*trans*	4.1	178.3	0.63
	cis/*cis*	0.0	0.0	1.31

Table S2: Excitation energies and oscillator strengths for the S_0 to S_1 absorption. Relative values are given with respect to conformation with the lowest the ground state energy.

Conformation	S_0-S_1 E [eV]	S_0-S_1 λ [nm]	ΔE [eV]	Δλ [nm]	osc.
**HS-84 *trans*/*trans***	2.75	451.1	0.00	0.0	1.92
**HS-84 *cis*/*trans***	2.80	442.6	0.05	-8.5	1.82
**HS-84 *cis*/*cis***	2.87	432.0	0.12	-19.1	1.59
**HS-167 *trans*/*trans***	2.71	457.9	0.23	-42.6	1.39
**HS-167 *cis*/*trans***	2.58	480.4	0.10	-20.1	1.44
**HS-167 *cis*/*cis***	2.48	500.5	0.00	0.0	1.42
**HS-169 *trans*/*trans***	2.37	522.1	0.00	0.0	1.24
**HS-169 *cis*/*trans***	2.35	528.1	-0.03	6.0	1.30
**HS-169 *cis*/*cis***	2.32	535.5	-0.06	13.4	1.28

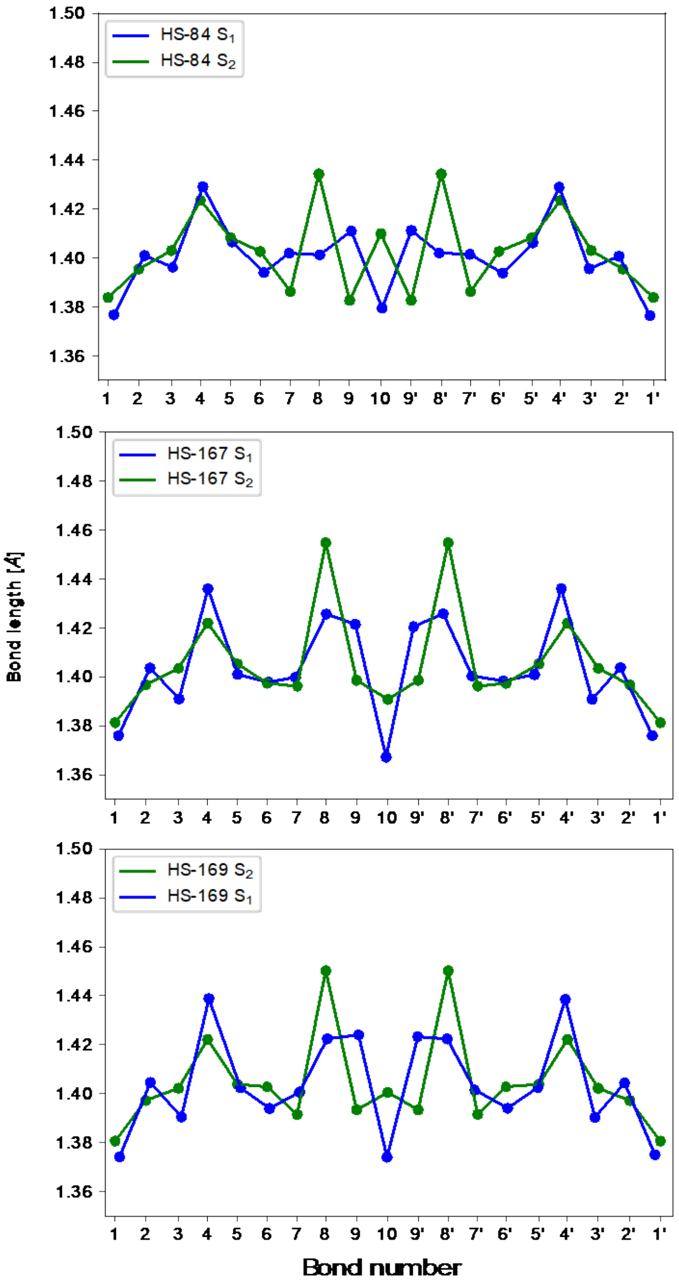

Figure S4. Bond length alternation pattern of the HS-84 (*trans*/*trans*), HS-167 (*cis*/*cis*) and HS-169 (*trans*/*trans*) conformations in first excited singlet state (S_1, blue) and first excited singlet state (S_2, green). For atom labels, see Figure 6.

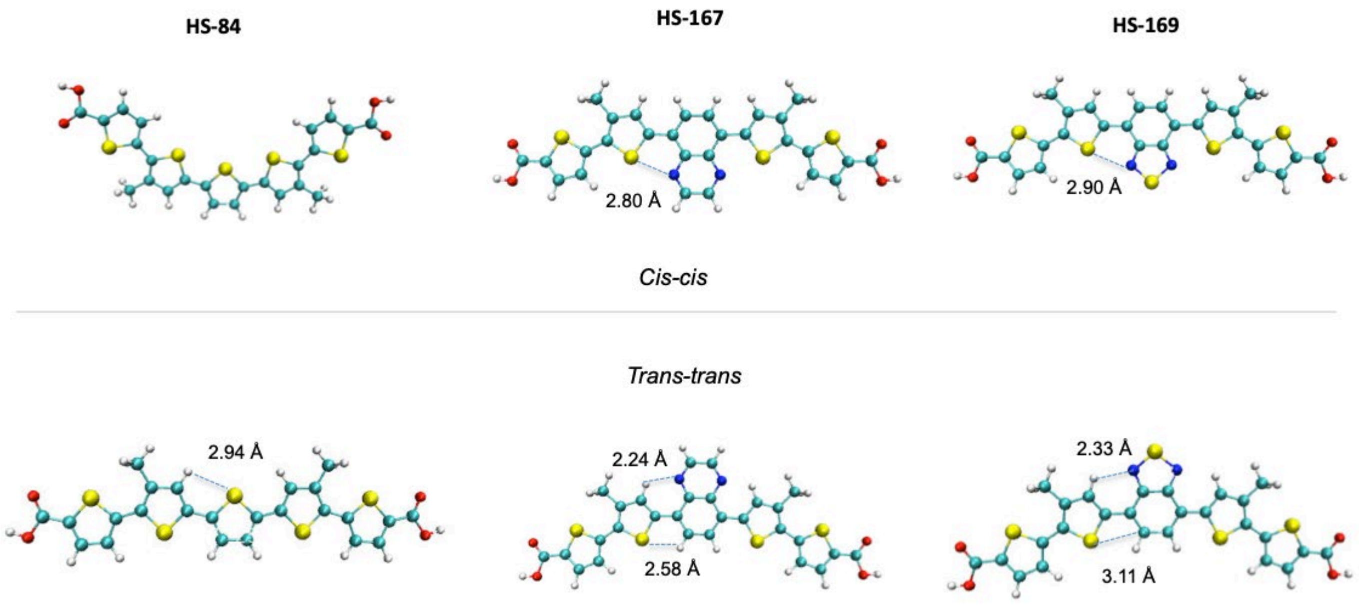

Figure S5. Stabilizing interactions in the planar *cis-cis* and *trans-trans* conformations of each biomarker.

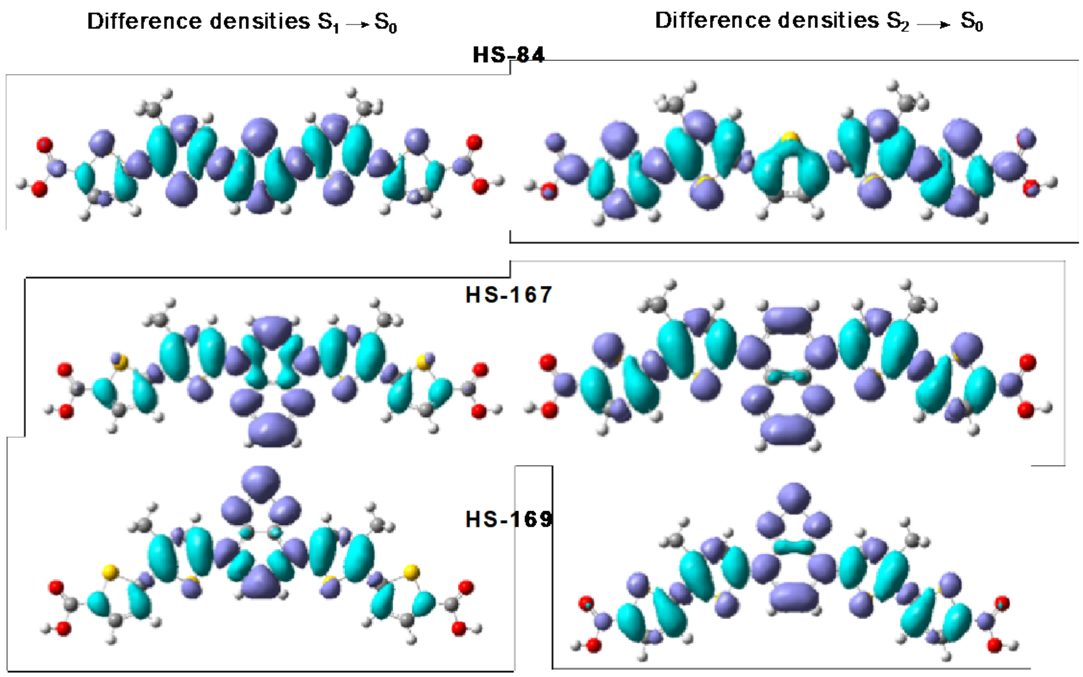

Figure S6. Detachment (cyan) and attachment (purple) densities for the first excited singlet state (S_1, left) and second excited singlet state (S_2, right).
